# Mentoring in Pediatric Thoracoscopy: From Theory to Practice

**DOI:** 10.3389/fped.2021.630518

**Published:** 2021-02-16

**Authors:** Francesco Macchini, Ernesto Leva, Valerio Gentilino, Anna Morandi, Steven Scot Rothenberg

**Affiliations:** ^1^Department of Pediatric Surgery, Fondazione IRCCS Ca' Granda Ospedale Maggiore Policlinico, Milan, Italy; ^2^Department of Clinical Sciences and Community Health, University of Milan, Milan, Italy; ^3^Department of Pediatric Surgery, Ospedale Filippo Del Ponte, Azienda Socio Sanitaria Territoriale Sette Laghi, Varese, Italy; ^4^Rocky Mountain Hospital for Children, Denver, CO, United States

**Keywords:** thoracoscopy, training, mentoring, teacher education, congenital lung malformation, esophageal atresia

## Abstract

**Introduction:** Thoracoscopy represents the most challenging area of pediatric minimally invasive surgery due to its technical difficulty. A standardized training program would be advisable. The aim of this study is to evaluate the results of our surgical training.

**Materials and Methods:** A retrospective, single-center, cohort study was performed. The following four-step program was tested: (1) theoretical part; (2) experimental training; (3) training in centers of reference; (4) personal operative experience. Particular attention was focused on the choice of mentor. Times and modality of adherence to the program were evaluated. The effectiveness and safety of the training were evaluated according to the surgical results of esophageal atresia (EA/TEF) repair and resection of congenital lung malformations (CLM). The study was conducted from January 2014 to May 2020. Attending surgeons with previous experience in neonatal and pediatric laparoscopy were selected for the training program after being evaluated by the head of Department.

**Results:** The training program was fully completed in 2 years. Twenty-four lobectomies, 9 sequestrectomies, 2 bronchogenic cyst resections and 20 EA/TEF repair were performed. Thoracoscopy was always feasible and effective, with no conversion. The operative times progressively decreased. Only three minor complications were recorded, all treated conservatively.

**Conclusions:** A standardized training program is highly desirable to learn how to safely perform advanced pediatric thoracoscopy. The 4-steps design seems a valid educational option. The choice of the mentor is crucial. An experience-based profile for pediatric surgeons who may teach thoracoscopy is advisable.

## Introduction

After the extensive diffusion of minimally invasive surgery (MIS) in the adult population, this surgical approach gradually involved the more challenging pediatric world (1).

In the last two decades MIS extended to an ever-growing number of indications in pediatric surgery and became the gold standard for certain diseases (2).

Among different applications of MIS in childhood, thoracoscopy is the most challenging as the thorax was the last area to be approached by MIS pediatric surgeons. Thoracoscopy is currently regarded by many pediatric surgeons as the last step in MIS training (1). In fact, it is technically more difficult than open surgery and requires a stepwise learning curve (LC). In particular, two factors are currently implicated to achieve competence: first case volume, but frequencies are typically low in pediatric surgery, so that a significantly longer time is required to reach a plateau of competence. Secondly, a standardized pediatric thoracoscopic training program does not exist yet (2, 3). As a consequence, only a limited number of pediatric surgeons are currently experts on this topic.

The recent development of instruments that are suitable for children has been crucial to making thoracoscopy feasible also in neonates and infants, so that even difficult procedures, such as esophageal atresia/tracheo-esophageal fistula (EA/TEF) repair and resection of congenital lung malformation (CLM), can be safely performed thoracoscopically (4).

In 2015, the European Society of Pediatric Endoscopic Surgeons (ESPES) published the guidelines for training program in pediatric minimally invasive surgery, recommending a four-steps training program (2).

The aim of our study is to evaluate the results of the surgical training in pediatric thoracoscopy in our Center, since the establishment of a new thoracoscopic program.

## Materials and Methods

We conducted a retrospective, single-center, cohort study at the Pediatric Surgery Department, Fondazione IRCCS Ca' Granda Ospedale Maggiore Policlinico in Milan, Italy. Parents gave written informed consent for surgical procedures and for the publication of this series in accordance with the Declaration of Helsinki. In 2012 we started a thoracoscopic training program.

The adherence of our training program to the ESPES guidelines for training program in pediatric minimally invasive surgery was evaluated, with the focus to verify the completion of all of the steps and the collection of the number of required procedures. In addition the surgical outcomes of our procedures were analyzed and also compared to the results of open surgery in our Center.

According to ESPES guidelines, a valid MIS training curriculum is represented by the “Peyton's Four-Step Approach” consisting in (5):

1. Theoretical part: attendance of at least 1 theoretical course in thoracoscopy;
2. Experimental training (MIS trainer, animal models, 3-D *ex vivo* models): spending at least 10–20 h of training on a pelvic trainer and at least 10 h of training on an animal model;
3. Training in a center of reference for pediatric MIS: attendance of 1–3 months of training in a center with high volume of MIS activity;
4. Personal operative experience: performing at least 30 procedures as assistant controlling the camera and more than 50 basic procedures as primary surgeon helped by a tutor (2).

### Training Curriculum

Only attending surgeons with previous experience in neonatal and pediatric laparoscopy were selected for the training program after being evaluated by the head of department. The training process started with the delivery of educational material and attendance to national and international courses (6). High-fidelity simulation sessions were performed according to two different modalities. The “dry lab” was performed with the use of traditional and high-fidelity MIS trainers (7, 8). Next hands-on training was delivered through “wet labs,” animal-based simulation sessions (9). An observership program at an international high volume referral center for pediatric thoracoscopy was the subsequent step (7). Professor S.S. Rothenberg from the Department of Pediatric Surgery of the Rocky Mountain Hospital for Children, Denver, Colorado (USA) was chosen as reference surgical mentor in view of his universally recognized experience in pediatric thoracoscopy (10, 11). Department directed by Prof. S.S. Rothenberg is a high-volume training center (>200 MIS procedures per year) (2) and trains pediatric surgeons from all over the world during the whole year. The first cases and the wider series of EA repairs and CLM resections come from this center, that published some of the most recent and updated papers (10, 11).

The next step consisted of performing a series of selected cases at our Hospital with the mentoring of Rothenberg (11). The first cases were performed by him with the training surgeons acting as first assistant. The following cases were managed by the local surgeons under his assistance and guidance. The last step was for the local team to manage the cases independently. For complex cases, pre-operative advice from the mentor was always required, by sharing through internet/web the history and images of patients. Patient's data were treated according to Data Protection Act.

The second end point of our study was the evaluation of the effectiveness and safety of our training according to our surgical results. This target was achieved focusing on two of the most difficult thoracoscopic procedures in pediatric surgery: EA/TEF repair in newborns and CLM resection during infancy (4). No modifications were made to the standard of care for newborns and infants requiring thoracoscopy for the studied diseases. Regarding EA/TEF, criteria for the thoracoscopic approach were: suspected standard type C EA/TEF, birth weight ≥2,000 g, absence of major associated malformations, and cardio-respiratory stability (no medical cardiac support and no mechanical ventilation). Reasons for conversion from thoracoscopy to open thoracotomy were any intraoperative adverse event or lack of progress for more than 15 min (12–14). According to the last step of our training program, the techniques recommended by Prof. Rothenberg in his last publications were selected as procedures of choice (10, 11).

Two additional aspects of our general approach deserve to be mentioned.

First, all cases are preoperatively evaluated by a multidisciplinary dedicated team, consisting of neonatologist, radiologist, pneumonologist, surgeon, anaesthesiologist. Images are collectively evaluated, with 3D reconstructions when possible (15).

Secondly, the surgical team, including anesthetists and scrub nurses, always performs a preoperative meeting and a post-operative critical analysis of the procedures. During the first one, the surgical anatomy and the technical steps are revised in details, with the support of internal schemes and videos, e-learning (www.websurg.com; www.globalcastmd.com), and videos from the mentor. In the post-operative meetings, one soon after surgery and one a month later, an evaluation of the entire procedure and of the related criticisms and complications are carried out. Surgical criticisms are also evaluated on the basis of the analysis of video recordings of the procedures.

### Surgical Outcome

To evaluate the impact of the surgical expertise on pediatric thoracoscopy, we retrospectively analyzed our case-series from the beginning of our surgical activity (January 2014) to present (May 2020). Only patients operated independently from the tutor were analyzed. We retrieved the following data: indications to thoracoscopy and demographic data; number of procedures performed with success; number of procedures needing conversion and reasons; length of surgery; perioperative, and postoperative complications.

A further comparison between the results of the thoracoscopic approach and the “open” traditional one was performed too, focusing on length of surgery and perioperative and postoperative complications. The population operated with the open technique was chosen among patients with characteristics similar to the thoracoscopic group, in terms of patients' age, indications for surgery and study period. It deserves to be specified that the open interventions were performed by a larger group of trained surgeons, including the surgeons selected for the thoracoscopic training.

The severity of every complication was assessed according to the Clavien-Dindo Classification of Surgical Complications (Table 1). It is a morbidity scale based on the therapeutic consequences of complications, that constitutes a simple, objective, and reproducible approach for comprehensive surgical outcome assessment (16).

**Table 1:** Classification of surgical complications grades definition (modified from Clavien-Dindo Classification).

Grade I	Any deviation from the normal postoperative course without the need for pharmacological treatment or surgical, endoscopic, and radiological interventions. Acceptable therapeutic regimens are: drugs as antiemetics, antipyretics, analgesics, diuretics, and electrolytes and physiotherapy. This grade also includes wound infections opened at the bedside
Grade II	Requiring pharmacological treatment with drugs other than such allowed for Grade I complications. Blood transfusions and total parenteral nutrition are also included
Grade III	Requiring surgical, endoscopic, or radiological intervention Grade III-a: intervention not under general anesthesia
Grade III-b	Intervention under general anesthesia
Grade IV	Life-threatening complication (including CNS complications)^‡^ requiring IC/ICU-management
Grade IV-a	Single organ dysfunction (including dialysis)
Grade IV-b	Multi-organ dysfunction
Grade V	Death of a patient

*^‡^Brain hemorrhage, ischemic stroke, subarachnoidal bleeding, but excluding transient ischemic attacks (TIA); CNS, Central Nervous System; IC, Intermediate care; ICU, Intensive care unit*.

The analysis of the results in terms of operative times and complications was limited to procedures with the higher number of patients and with the relatively lower variables, i.e., lobectomies, sequestrectomies and EA/TEF repairs.

Variables were expressed as mean ± standard deviation (SD) for demographic and baseline variables, and as median and range for length of follow-up. The learning curves were analyzed using linear regression.

Parametric variables were compared with the Student's *t*-test. Categorical variables were evaluated by Fisher exact test. Statistical analysis was performed using SigmaStat® (Systat Software Inc., San Jose, CA, USA). A *p* < 0.05 was considered significant.

## Results

### Training Curriculum

Two surgeons previously trained in neonatal and pediatric laparoscopy and with experience in basic thoracoscopic procedures were selected. As regards laparoscopy, Nissen fundoplication, extra-mucosal pyloromyotomy, appendectomy, cholecystectomy, and surgery for Hirschsprung disease, were the most common and well-known performed procedures. Regarding previous thoracoscopic experience, neonatal removal of thoraco-amniotic shunts dislodged in thorax, treatment of pleural empyema and lung biopsies have been performed in a satisfactory number of patients. In particular, selected surgeons had a previous personal experience in more than 500 neonatal and pediatric minimally invasive procedures. The four-step training program was fully completed in 2 years (June 2012–2014).

Here following the details of every single step are reported.

- Theoretical knowledge: 4 theoretical national and 2 theoretical international courses were attended by the selected surgeons. All courses were focused on minimally invasive thoracic surgery and were organized under the sponsorship of national, European and international societies.
- Practice-based learning and improvement in experimental setting: 1 national and 1 international “dry lab” courses were attended, using both traditional and high-fidelity MIS trainers. Simulations were performed systematically even after treating the first patients in order to maintain a high level of technical ability. One national and 1 international “wet lab” courses were attended, both using young anesthetized piglets. During dry and wet labs, thoracoscopic lobectomies and esophageal anastomosis were performed. These steps were further developed by attending the post-graduate Master program in Pediatric Minimally Invasive Surgery at Alma Mater Studiorum—Università di Bologna—Italy.
- Stages in centers of reference: as previously described, a 15 day observer-ship was completed at the Department of Pediatric Surgery of the Rocky Mountain Hospital for Children, Denver, Colorado (USA), under the mentoring of Professor S.S. Rothenberg. During this period many thoracoscopic procedures were observed, such as pulmonary lobectomies, sequestrectomies, closures of patent ductus arteriosus, bronchogenic and pleural cysts resections, corrections of pectus excavatum.
- personal operative experience: within 6 months after the observership in USA, the first 10 cases of CLM were operated on as assistant with Professor S.S. Rothenberg working as main surgeon. In particular, trainees operated 5 pulmonary lobectomies, 1 bronchogenic cysts and 1 extra-lobar pulmonary sequestration as first assistant. The series included also 1 closure of patent ductus arteriosus and 1 esophageal duplication. Then, 2 pulmonary lobectomies and a resection of bronchogenic cyst were operated as first surgeon helped by the mentor. Finally the thoracoscopic procedures were approached without the presence of the tutor. In cases with challenging malformations, images, and advices were shared by web the days before. The first neonatal thoracoscopy for EA/TEF correction was performed after almost 1 year since the first thoracoscopic CLM resection. Due to the urgent need for surgery, typical of this malformation, the procedure was done without the mentor. Also in this case images and videos were shared before surgery. As for the personal experience, dedicated surgeons were selected among those highly trained in neonatal and pediatric surgery and with previous high experience in minimally invasive pediatric surgery. At the end of the training period, more than 30 thoracoscopic procedures were performed as cameraman and more than 80 as primary surgeon.

### Surgical Outcome

As regards the 2 thoracoscopic procedures selected for the present study, 35 CLM resections and 20 EA/TEF repairs were performed as main surgeons in the study period in Fondazione IRCCS Ca' Granda Ospedale Maggiore Policlinico in Milan, Italy.

**Figure 1:**
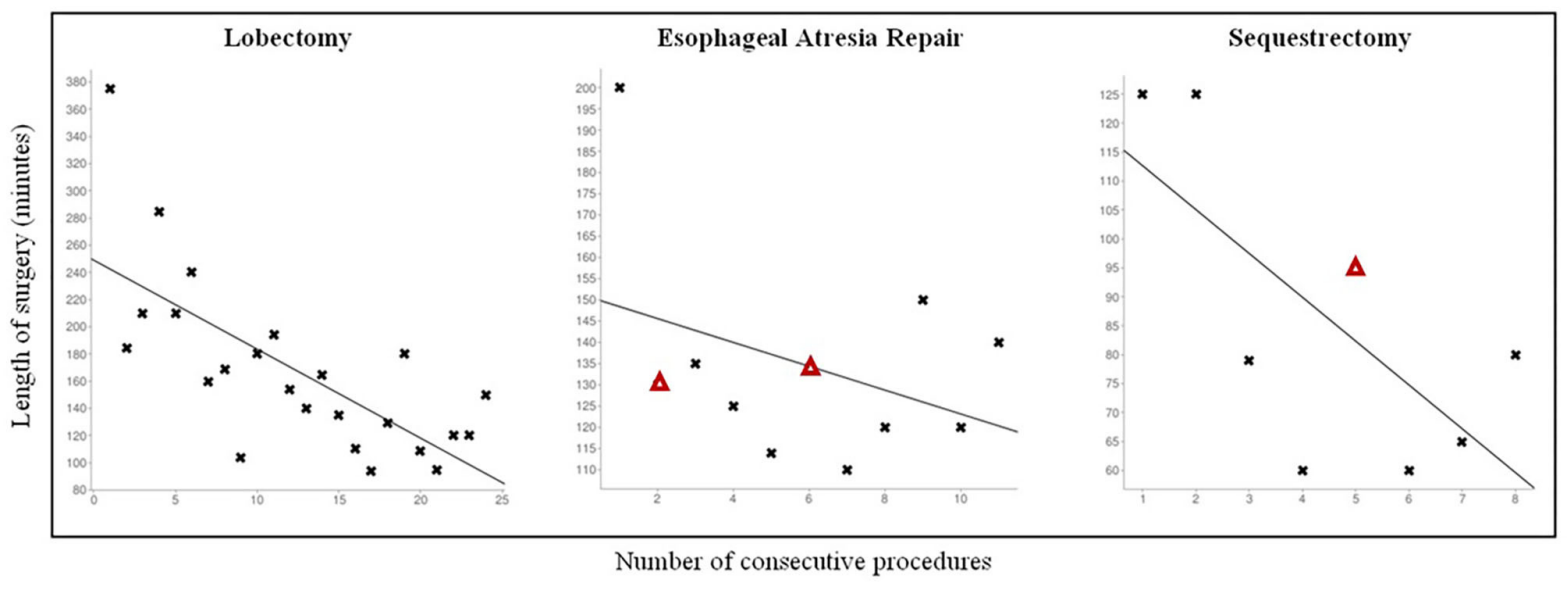
Personal learning curve for different pediatric thoracoscopic procedures. **Δ**: Patients with Grade II complication according to Clavien-Dindo Classification.

The CLM population received the following operations: 24 lobectomies, 9 sequestrectomies, and 2 bronchogenic cyst resections. Mean age at surgery was 7.6 ± 3.3 months, 19 patients were males. Thoracoscopy was feasible and effective in all cases, no conversion was observed. No major anatomical variants were detected in patients where a 3D CT scan reconstruction was possible. This data was consistent with the intraoperative findings. Mean operative time was 167 ± 65 min for lobectomies and 84 ± 27 min for sequestrectomies. As regards lobectomies, a significant reduction in operative times (*p* = 0.0001) was observed while increasing the surgical experience (Figure 1). Also operative times of sequestrectomies progressively reduced, though not reaching statistical significance probably due to the small number of patients (*p* = 0.21).

Regarding the comparison between traditional and MIS surgery, in the same study period 34 lobectomies, 2 sequestrectomies, and 2 bronchogenic cyst resections were performed in our Center with a thoracotomic approach. Mean operative time was 163 ± 45 min for lobectomies (*p* = 0.83). The mean surgery length was 110 min for sequestrectomies and 100 min for bronchogenic cyst resections but the limited numbers of both populations don't allow a statistical analysis.

The EA/TEF population consisted of 20 patients (13 males). Eleven newborns (55%) had a distal TEF (type C according to Gross classification), 5 (25%) an isolated TEF (type E), 3 (15%) had no fistulas and were classified as long gap (type A), and 1 (5%) proximal and distal fistulas (type D). The satisfactory results obtained with the first 5 EA/TEF cases prompted us to treat long gap forms too.

For EA with distal TEF (type C), mean gestational age at surgery was 38 ± 1.6 weeks, mean age was 1.7 ± 1 days and mean weight 2.763 ± 466,6 g.

Thoracoscopy was feasible and effective in all cases. No need for conversion was observed. Mean operative time was 134 25 min. The operative times rapidly decreased after the first case, though not reaching statistical significance (*p* = 0.67) probably due to the small number of patients, and remained stable for the subsequent procedures.

In the study period 18 type C EA were traditionally approached. Mean operative length was 126 ± 34 min (*p* = 0.07).

A total of 3 patients with a long gap malformation were operated on thoracoscopically. MIS was effective in performing a direct esophageal anastomosis in all cases and no conversion was recorded.

Regarding the MIS post-operative period, only 1 CLM patient (3%) developed an early complication. One male with an extralobar sequestration, that was prenatally ablated, had a post-operative bleeding that resolved after tranexanic acid iv therapy, probably as a consequence of the extensive blunt adhesiolysis between the lung and the ES (Grade II according to Clavien-Dindo Classification).

Two damages to the middle lobe requiring additional resections (Grade III b) occurred in the thoracotomic group.

Within C-type EA repair, 2 minor leakages were observed in both groups (MIS 18% vs. Open 11%; *p* = 1). Patients were treated conservatively with i.v. antibiotics and fasting, and presented a spontaneous resolution of the leakage. These complications were classified as Grade II according to Clavien-Dindo Classification. Six (50%) MIS vs. 9 (50%) open patients developed an anastomotic stricture (*p* = 1) requiring dilations (mean ± DS number of dilations: MIS 0.83 ± 1.0 vs. Open 0.94 ± 1.2; *p* = 0.4). These complications were classified as Grade IIIb according to Clavien-Dindo Classification. No significant differences in the prevalence of gastro-esophageal reflux disease was observed between the two studied groups (MIS 3/12–25% vs. Open 7/18–39%; *p* = 0.69).

As previously reported, all procedures were analyzed during the multidisciplinary post-operative meeting, where an evaluation of the entire procedure and of the related criticisms was done, and the follow-up was revised during the monthly meeting.

The mid-term follow-up was uneventful too. At follow-up (median time 24 months, range 1–46) children with CLM resection are clinically and radiologically healthy. Children with EA/TEF repair at a median follow-up time of 30 months (range 3–39) are clinically in good health. From an endoscopic point of view, 5 children (45%) developed a post-anastomotic stenosis that was successfully treated in the first 6 months of life with a mean of 1.8 ± 0.8 pneumatic dilations. In our series, the incidence of esophageal post-anastomotic stenosis is similar to the population operated with an open technique and to experiences from other centers (12, 17).

## Discussion

In the last few decades, MIS has significantly developed, extending to infancy and childhood. This progress was also due to the introduction of advanced techniques and specific devices for the management of little spaces and little anatomical structures.

However, among the different applications of MIS in childhood, thoracoscopy represented the last area to be reached. Although thoracoscopy represents a well-established approach for infants and children, reducing pain and morbidity and avoiding the long-term consequences of a thoracotomy (11), it is not still widely adopted, due to the fact that it is technically more difficult under different aspects. Main difficulties are due to anatomical features in children: pleural and mediastinal areas are minimal; intercostal spaces are limited, especially in neonates, not allowing the use of instruments >5 mm; the anatomy may be distorted by the original malformation; manipulated structures are thin and eventual complications may be fatal (1).

Technical aspects are involved in the delay in pediatric thoracoscopy too: the discrepancy between limited operative spaces and the large size of the available instruments, with a length and thickness of the jaws often disproportionate to the operative space of the infant (4). In addition, case volume and frequency are typically low in pediatric surgery, and a significantly longer time is required to reach competence. Finally, capnothorax is not always well-tolerated by neonates and infants, further reducing the number of procedures (18).

The final difficulties concern training. In many regions worldwide the training in pediatric surgery does not include a fellowship in adult cardio-thoracic surgery. Moreover, a standardized pediatric thoracoscopic training program does not exist yet (3).

Recently, the European Society of Pediatric Endoscopic Surgeons (ESPES) proposed guidelines for an European MIS training program for pediatric surgeons, with the aim to construct a curriculum that provides a safe, uniform, efficient and procedure-specific training program to gain experience, while maintaining patient safety. ESPES hoped that European countries would adopt this program so as to secure a standardized technical qualification in MIS for all future pediatric surgeons (2).

We tried to reproduce the suggestions by ESPES in our training program in pediatric thoracoscopy. The four-steps training program was fully completed in 2 years.

All steps reached high levels of satisfaction from the participants.

The basic knowledge of MIS procedures by the training surgeons was an important starting point, thanks to the acquired knowledge in handling thin structures and operating in limited spaces as in neonatal and infant thoracoscopic and laparoscopic procedures, as well as in doing intracorporeal knots. This condition allowed to overcome the mentioned limits of pediatric MIS training, especially due to low volume and frequency of cases.

In our personal experience, the theoretical step represented a significant occasion to go deep into the topic.

According to the participants, the practice-based learning appeared more similar to reality and crucial from a surgical point of view to develop adequate technical skills.

Previous steps reached a high level of interest and utility, but we think that a special notice has to be given to the choice of the mentor that has to be highly accurate. In fact it represents not only the step immediately before the starting of surgery, but also the on-going support when the trained surgeons begin to operate independently.

We think that some points about this step need to be critically analyzed.

The first point is how to choose the mentor. At the moment there are a lot of training centers for MIS, but standardized essential requirement to certify them still does not exist. As suggested by ESPES guidelines, a training center should be able to offer a complete MIS training, with structured “dry” and “wet” laboratories. Trainees should participate in complex surgery and undergo structured training in all aspects of the procedure and then perform a designated number of cases under supervision (2).

In our experience some points of mentoring revealed crucial: to study the tutor's technique, reading his papers and watching the videos of his operations; to see and help him as assistant, trying to learn his tips and tricks; to operate with him as primary surgeon, following his live suggestions; to keep in strict connection even after the period of training. As regards tips and tricks, our mentor recommended us to introduce the use of miniaturized instruments, especially designed for neonates and infants. In our experience, these new instruments facilitated the thoracoscopic procedure and helped us to reduce the length of surgery (4).

As a general consideration, our experience shows that the surgical approaches suggested by Prof. S.S. Rothenberg are safe and reproducible, after an appropriate training. Results similar to other international centers (12) are achievable in a relatively short time frame.

Another important contribution to this last aspect may come from tele-mentoring: to overcome the lacking continuity in oversight by instructors, a remote teacher–student interaction is established through the web in different ways. Instructors use the connection to assess video recorded training sessions of students at distant locations and guide them through the MIS procedures with specific and personalized feedback. Tele-mentoring may be as effective as in-person instruction in teaching advanced MIS surgical skills, providing an effective method of teaching remotely and allowing expansion of robust simulation training curricula (19). We never adopted this tool but we think it may be very useful and will develop significantly in a near future.

According to all these considerations, we think that a step should be added to the training course, specifically dedicated to the choice and modalities of mentoring.

The results of our training program seem promising. Considerable numbers of thoracoscopic CLM resections and EA/TEF repairs were effectively performed in a relatively short period. Despite being still in training, the length of surgery did not significantly differ from the open group, thus not increasing the surgical and anesthesiological patient's exposure.

As regards the patients' safety, no major intra- and early post-operative complications, such as complications requiring surgical, endoscopic or radiologic intervention, life-threatening complications or death (16), were recorded. Focusing on CLM resection, the open group presented two major complications requiring additional surgery. One possible explanation of these results may be related to the magnification provided by the thoracoscopic approach that helps in a better anatomical vision. As regards EA/TEF repairs, no significant differences were recorded between the two groups, both in early and in late complication rate.

Pediatric thoracoscopy represents a fascinating and innovative technique, encountering in our experience a high level of satisfaction both for families and for the whole team.

The traditional “Master–Apprentice Model” revealed useful in the first part of our training, allowing us to reach the basic knowledge and technical competences to perform thoracoscopy in children. In this model, the apprentice first learns to perform a procedure by observing the master or surgeon how it needs to be done; then the trainee has to assist the surgeon several times; finally he will gradually be allowed to perform parts of the operation under the master's supervision until the apprentice can eventually perform it by himself (2, 20). The collaboration with the mentor in our experience also led to research projects and the organization of international workshops on pediatric thoracic surgery in 2017 and 2019.

In summary, in our experience a multistep training program as previously described seems effective to start pediatric thoracoscopy safely. A support to this consideration also comes from results we obtained following the same steps in other surgical areas, such as the fetoscopic correction of myelomeningocele (21) and the setting of a neonatal ECMO team (22).

We think that some further points of our method concurred to provide adequate surgical skills for an effective execution of thoracoscopy, especially in neonates and infants. A dedicated team including anesthetists, neonatologist, radiologist, pneumologist, and nurses, should be selected at the beginning of the training program and all steps should be shared with all the team. A multidisciplinary approach ensures a detailed preoperative assessment; a specific neonatal stabilization and study of the child; an appropriate intra-operative management; and a right post-operative and long-term follow-up.

A preoperative meeting and a post-operative critical analysis of the procedures are valuable. During the first one, the surgical anatomy and the technical steps are revised, and the intervention is planned in details. In our experience, an important help in deepening the anatomy and any anatomical variants of the lungs is guaranteed by the CT-scan images and, when possible, by the use of 3D reconstructions. In fact, as reported by previous studies (23), variations in lung anatomy are frequent and the benefits of a precise pre-operative evaluation are currently stressed by many authors. In the post-operative meeting, the evaluation of the entire procedure and of the related criticisms and complications are always an opportunity to make steps forward. A significant help to this came in our experience from the systematic recording of the procedures, that were subsequently always reviewed and analyzed. This last step revealed useful also in our hospital to organize local and international meetings on this specific topic.

A last issue may be of some help in reducing the problem of low case volume and frequency and the subsequent slowness of the LC. Whenever possible, the opportunity to attend the operatory rooms of adult thoracic surgery may be useful for a constant revision of anatomy. Although the approaches in adult surgery are different from the pediatric ones, a significant improve may come from their experience and high volume activity. In addition, a centralization of the studied malformations in high level centers should be advisable. CLM and EA are considered rare diseases and need a dedicated training with high skill levels to provide the best management and outcome. Centralizing these patients would ensure higher volumes and accordingly better expertise.

After the completion of the described process, since 2018 the same training program involving other surgeons and trainees has started, being mentored by the already skilled local surgeons.

We think that the main limitation of the present study is represented by being a single center observational study. Despite this aspect provides uniformity of the studied population, we strongly advocate further multicentric studies to verify the reproducibility and safety of our training program.

## Conclusions

Pediatric thoracoscopy represents the most challenging area of MIS. A standardized and certified training program is advisable. The 4-steps training seems a valuable educational proposal. The choice of the mentor is crucial.

## Data Availability Statement

The raw data supporting the conclusions of this article will be made available by the authors, without undue reservation.

## Author Contributions

FM contributed conception and design of the study. FM and VG collected and analyzed the data retrospectively. FM, AM, EL, and SR wrote the first draft of the manuscript. All authors contributed to manuscript critical revision, read, and approved the submitted version.

## Conflict of Interest

The authors declare that the research was conducted in the absence of any commercial or financial relationships that could be construed as a potential conflict of interest.
